# A novel system based on artificial intelligence for predicting blastocyst viability and visualizing the explanation

**DOI:** 10.1002/rmb2.12443

**Published:** 2022-02-07

**Authors:** Noritoshi Enatsu, Isao Miyatsuka, Le My An, Miki Inubushi, Kunihiro Enatsu, Junko Otsuki, Toshiroh Iwasaki, Shoji Kokeguchi, Masahide Shiotani

**Affiliations:** ^1^ Hanabusa Women’s Clinic Kobe Hyogo Japan; ^2^ NextGeM inc. Tokyo Japan; ^3^ Assisted Reproductive Technology Center Okayama University Okayama Japan

**Keywords:** artificial intelligence, assisted reproductive technology, gradient‐weighted class activation mapping, in vitro fertilization

## Abstract

**Purpose:**

The purpose of the study was to invent and evaluate the novel artificial intelligence (AI) system named Fertility image Testing Through Embryo (FiTTE) for predicting blastocyst viability and visualizing the explanations via gradient‐based localization.

**Methods:**

The authors retrospectively analyzed 19 342 static blastocyst images with related inspection histories from 9961 infertile patients who underwent in vitro fertilization. Among these data, 17 984 cycles of single‐blastocyst transfer were used for training, and data from 1358 cycles were used for testing purposes.

**Results:**

The prediction accuracy for clinical pregnancy achieved by a control model using conventional Gardner scoring system was 59.8%, and area under the curve (AUC) was 0.62. FiTTE improved the prediction accuracy by using blastocyst images to 62.7% and AUC of 0.68. Additionally, the accuracy achieved by an ensemble model using image plus clinical data was 65.2% and AUC was 0.71, representing an improvement in prediction accuracy. The visualization algorithm showed brighter colors with blastocysts that resulted in clinical pregnancy.

**Conclusions:**

The authors invented the novel AI system, FiTTE, which could provide more precise prediction of the probability of clinical pregnancy using blastocyst images secondary to single embryo transfer than the conventional Gardner scoring assessments. FiTTE could also provide explanation of AI prediction using colored blastocyst images.

## Graphical abstract

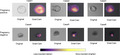

## 1 INTRODUCTION

The ability to predict embryo viability through assisted reproductive technology (ART) is important for both clinicians and patients, as it profoundly impacts embryo selection for transfer. Since the inception of human in vitro transfer (IVF), grading systems due to morphological characteristics using optical microscope have been developed by embryologists.^1^, ^2^ The Gardner scoring, which assesses morphological features such as inner cell mass and trophectoderm (TM) quality with embryo developmental advancement, has been most widely used all over the world.^3^ Recently, the clinical use of preimplantation genetic testing (PGT) has become widespread. This technology enables more accurate embryo selection by analyzing the embryo's chromosomes. However, PGT requires embryo biopsy to obtain embryonic genetic materials, thereby increasing the cost of IVF and the potential risk of compromising embryo viability in some cases.^4^ Moreover, recent studies have reported that some embryo classified as mosaic in PGT and therefore unable for transfer were reclassified as chromosomally normal in re‐analysis of PGT.^5^, ^6^, ^7^ Other researchers also suggested that the transfer of “abnormal” embryos (as per PGT) offered robust pregnancies and high chances of live births with low miscarriage rates; therefore, PGT cannot reliably determine which embryos should or should not be transferred.^8^ Therefore, the development of a new embryo grading technology that enables accurate prediction of embryo viability is still an important challenge.

Several studies have been conducted regarding testing a non‐invasive artificial intelligence (AI)‐based approach to aid in predicting embryo viability during IVF.^9^, ^10^ The reported accuracy of prediction is about 0.65, which indicates that AI models can improve the accuracy of prediction by 10%–20% compared with traditional grading methods (<50%).^9^, ^11^ Since there are lots of variables that affect pregnancy, including uterine condition, hormonal status, and complications besides infertility, the theoretical accuracy of predicting clinical pregnancy is estimated to be <80%.^12^ In fact, the success rate of clinical pregnancy of embryo transfer using euploid blastocysts is reported as about 70%.^13^ Therefore, a non‐invasive technology that can predict clinical pregnancy with 70% accuracy will have great value.

This study aimed to develop a novel AI system that can predict clinical pregnancy using ensemble modeling to combine blastocyst images and clinical data, such as age, hormonal status, and uterine condition.

Another goal of this study was to develop an explanation function regarding AI prediction. The black box mechanism of deep learning is considered a major hindrance for clinical application. For example, if an AI system predicts the viability of certain embryos and the predictive value was dissociated from the traditional grading system, it will cause some difficulties to physicians regarding explaining the result. Many methods—such as saliency mapping, class activation mapping (CAM), and gradient‐weighted CAM (Grad‐CAM)—have been developed for the visual explanation of AI systems.^14^ Grad‐CAM generates a heatmap that visualizes the class‐discriminative region. This will help physicians to identify regions of clinical value. This study developed a novel AI system for predicting blastocyst viability. We also investigated the validity of this system by regional visualization using Grad‐CAM.

## 2 MATERIALS AND METHODS

### 2.1 Study design

This study was based on a retrospective analysis which aimed to invent a novel AI system named Fertility image Testing Through Embryo (FiTTE) using 19 342 static day‐5 blastocyst images with related inspection histories from 9961 infertile patients who underwent IVF, including intracytoplasmic sperm injection, at Hanabusa Women's Clinic between January 2011 and August 2019.

### 2.2 Patients

This analysis included all patients who underwent single embryo transfer with known pregnancy outcomes. There were no exclusion criteria based on patient characteristics. Clinical data, including age, serum anti‐müllerian hormone (AMH), hormonal profiles, pregnancy history, ART history, height, weight, body mass index, menstrual cycle, blood pressure, endometrial thickness, and ART method, are available for 1358 patients. Since the ensemble AI model requires both patient inspection histories and hormonal profiles of cycles as inputs, the development of such models was restricted to this subset of 1358 embryos. All data were anonymized and sent to NextGeM Inc. for analysis. All patients were well‐informed regarding the use of these medical data for research purposes, and written informed consent was obtained from them prior to the treatment period. A Web site with additional information, including an opt‐out button for this study, was set up in the official website of Hanabusa Women's Clinic.

### 2.3 Control model using embryo grading by embryologist

AI algorithm based on conventional embryo grading by experienced embryologists was used as a control model. Conventional embryo grading system was based on Gardner's grading scale.^15^ Embryos were evaluated at day five or six after oocyte retrieval. Embryos that had not yet progressed pass the morula stage were excluded from this study. The Gardner's grading scales (grade 1, 2, 3, 4, AA, AB, CC, etc.) evaluated by the embryologists were introduced into the ResNet, a deep convulsion neural network, instead of the blastocyst images in FiTTE for the purpose of obtaining the predictive label of the control model. For the control model, a total of 96 690 cycles of single‐blastocyst transfer data were used for training, and 19 338 were used for testing purpose.

### 2.4 Image processing

**FIGURE 1:**
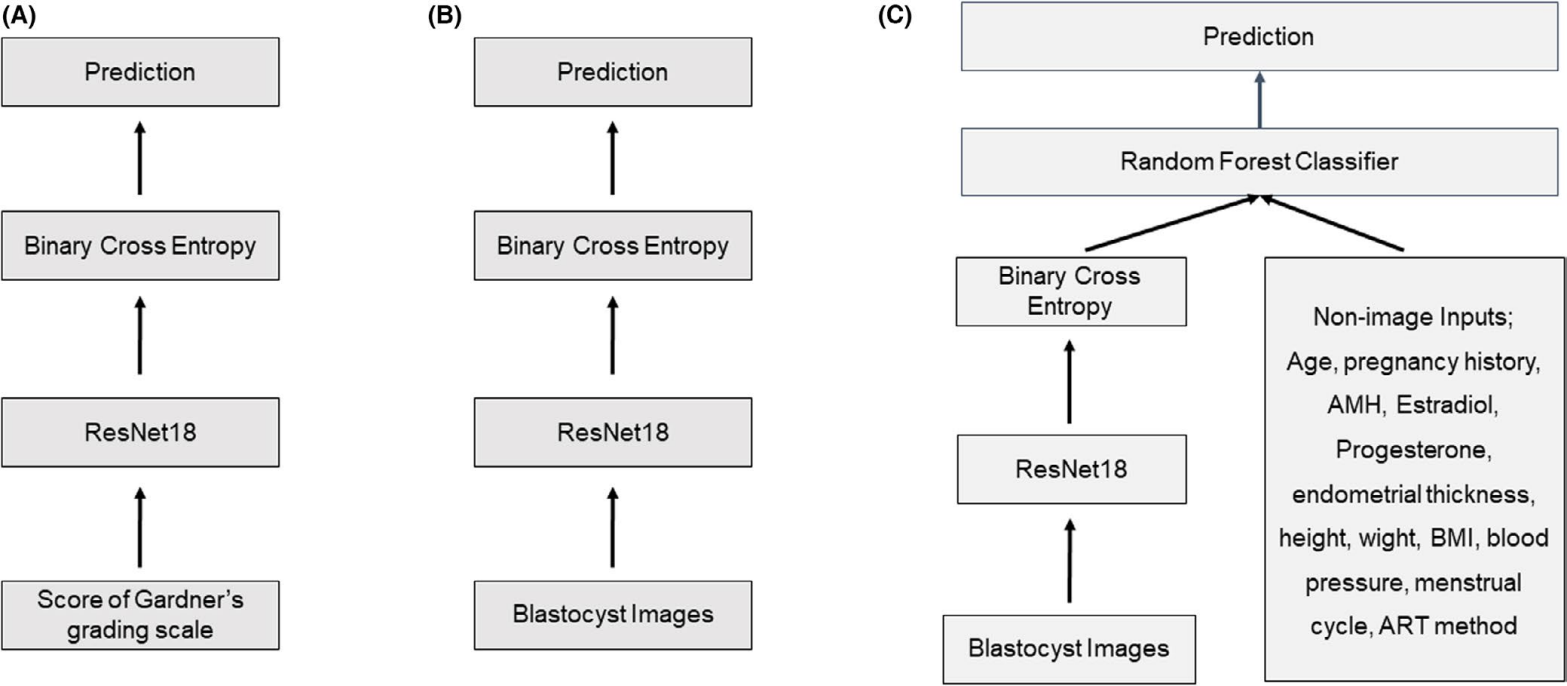
Layer algorithm from images and clinical data for a neural network for prediction of clinical pregnancy or live birth. In the image analysis, blastocyst images those resulted in positive serum human chorionic gonadotropin and fetal heartbeats were regarded as “viable’” and the other images were regarded as “non‐viable.” (A) Prediction algorithm from Gardner's grading scales evaluated by an embryologist (control model). (B) Prediction algorithm from blastocyst images (image‐only model; FITTE). (C) Prediction algorithm from the ensemble of blastocyst images and non‐image clinical data (ensemble model). Res Net; residual network. AMH; Anti‐mullerian hormone. ART; assisted reproductive technology

Images were converted into grayscale and rescaled into a resolution of 480 × 640 pixels. All images were initially labeled as “viable” or “non‐viable” according to the pregnancy outcome, in other words, blastocyst images those resulted in positive serum human chorionic gonadotropin (HCG) and fetal heartbeats were regarded as “viable” and the other images were regarded as “non‐viable.” Two types of algorithms were evaluated: an image‐only model and an ensemble model, which combines deep learning algorithms for image inputs and machine‐learning algorithms for non‐image inputs (Figure 1).

### 2.5 Outcomes

The primary end point of this study was clinical pregnancy as defined by a rising serum human chorionic gonadotropin (hCG) test and fetal heartbeats in the uterus as detected by transvaginal ultrasound. The secondary outcome was live birth. Although it is more important than clinical pregnancy, its sample size was smaller than that for clinical pregnancy, and the ensemble model for live birth prediction was not completed because of an insufficient sample size for deep learning; therefore, we set clinical pregnancy as the primary end point and live birth as the secondary outcome. Accuracy is used as the main measure to evaluate the performance of AI algorithms and is defined as the percentage of both viable and non‐viable embryos correctly identified by AI models. Accuracy is used as the main measure to evaluate the performance of AI algorithms and is defined as the percentage of both viable and non‐viable embryos correctly identified by AI models. Another measure for the performance of FiTTE was calculated using the receiver operator characteristic (ROC) curve generated by plotting the true positive rate against the false‐positive rate across all positive thresholding values using the predicted confidence score compared with the actual pregnancy outcome.

Another end point of this study was confirmation of the visualization model for explaining AI prediction via visualization algorithm Grad‐CAM to confirm the validity of the model.

### 2.6 Algorithm architecture and training methods

Figure 1 shows the layer algorithm of FiTTE, which represents the process from images and clinical data to predicting clinical pregnancy or live birth. For the prediction from blastocyst images, images were first set into residual network (ResNet18), which is widely recognized as a great learning model in the field of image recognition^16^ that offers a deep convulsion neural network. Convolutional neural networks (CNNs) comprise several convolutions to pass the result to the next layer, pooling layers to combine the outputs of neurons into a single neuron, and fully connected layers, which represent the outputs.^17^, ^18^ FiTTE utilizes five convolution blocks made of 18 layers, two pooling layers, and one fully connected layer. The architecture ends with binary cross entropy for prediction. In this study, we used the embryo image and non‐image clinical data from 17 984 cycles of single‐blastocyst transfer for training. Additional data from 1358 cycles were used for testing purposes. For live birth prediction, data from 10 643 cycles of single embryo transfer cycles were used for analysis. Among these cycles, 9091 were used for training, and 1552 were used for testing purposes. Figure 1B shows that a prediction algorithm from an ensemble of blastocyst images and non‐image clinical data consisted of the same algorithm from image process to binary cross entropy. The processed data were then set into random forest classifier with non‐image clinical data for prediction. Because of the limited sample size (1358) including those with all the required data, the predictive accuracy for the ensemble model was evaluated using the 10‐fold cross‐validation method.

### 2.7 Grad‐CAM (gradient‐weighted class activation method)

In order to visualize the explanation of AI prediction, we used the Grad‐CAM. It uses class‐specific gradient information flowing into the final layer of a CNN to produce a map of important regions in the image. The last convolution layer is the layer right before the final layer which generates class predictions. Consequently, the neurons in this last convolution layer should summarize which features in the image are important in making these predictions. For each prediction class, Grad‐CAM uses the gradient information flowing through the last convolution layer to assign importance values to each neuron.^14^

## 3 RESULTS

Of the 19 342 day‐5 blastocysts (7,717 resulted in clinical pregnancy with fetal heartbeat), 1358 embryos having all the required image and non‐image inputs were reserved for testing purposes. The remaining 17 984 images were split into training (~90%) and validation (~10%) sets for the development of an image‐only AI model (FiTTE). The ensemble AI model was trained using 1223 cases, and 135 cases were used for testing purpose. The live birth prediction was developed in the same algorithm using 10 643 blastocyst images. Among these data, 1552 were used for testing purpose. The ensemble model for live birth prediction was not completed because the sample size of 624 cases was not enough for deep learning.

**FIGURE 2:**
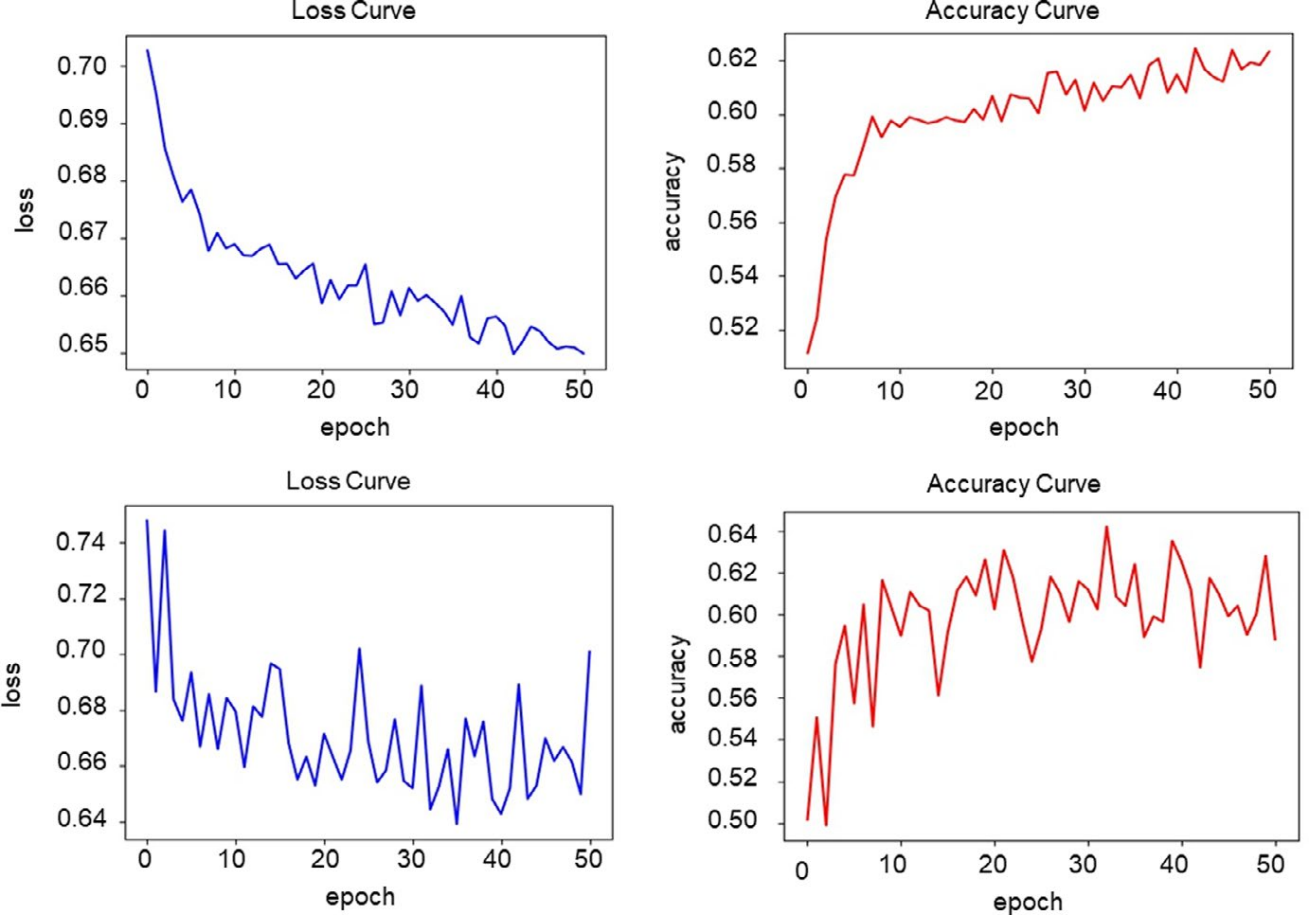
Performance in the training and validation curves for convulsing neural network models. Upper row: image‐only model. Lower row: ensemble model

Figure 2 represents the change in the accuracy and loss in the training and validation curves for the CNN models. Both the loss and accuracy curves converged to similar loss value in the upper row, indicating that the AI model trained without overfitting to the training data. The degree of convergence is not satisfied in the lower row, indicating an insufficient sample size in the ensemble model.

**FIGURE 3:**
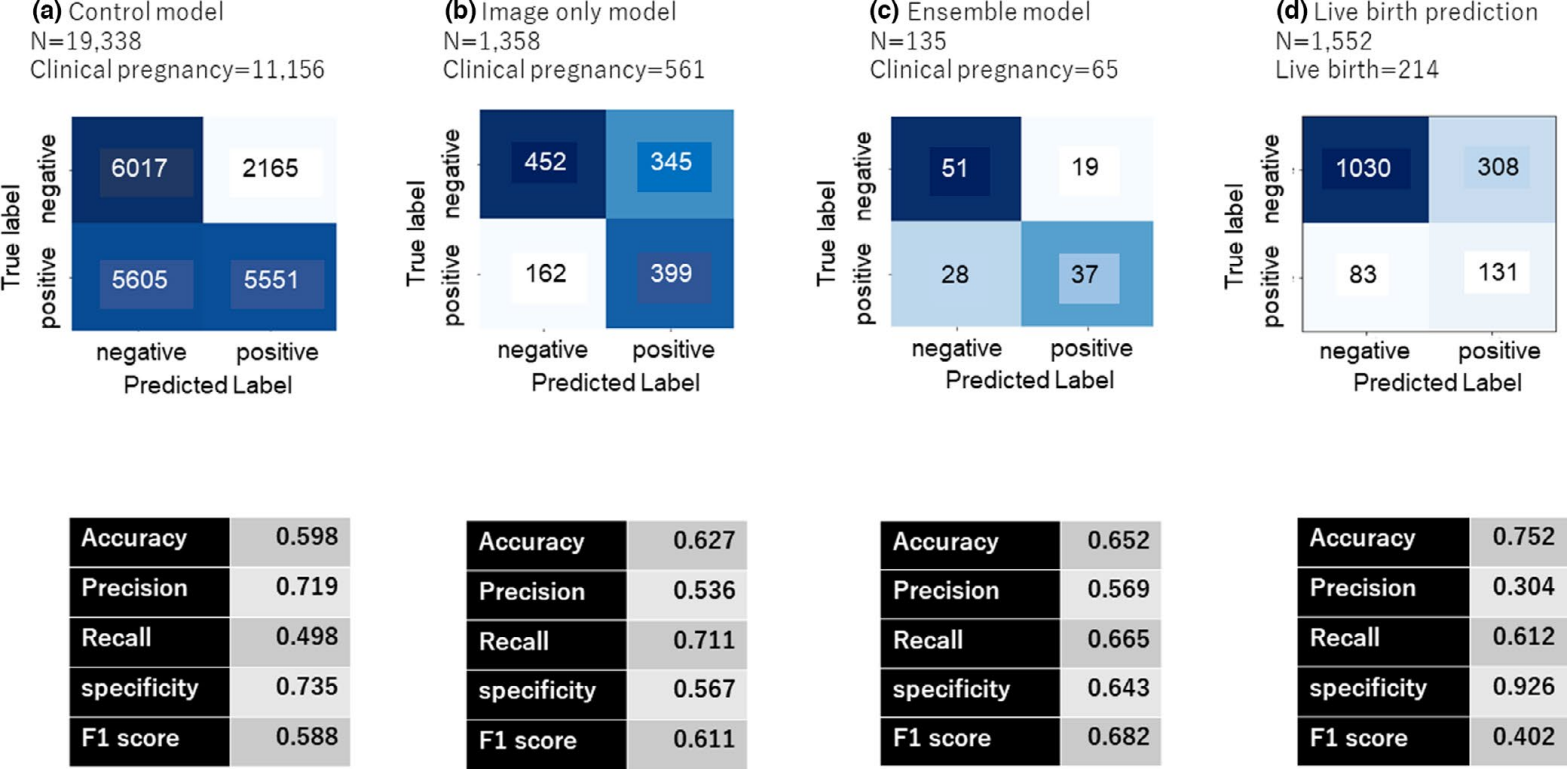
Confusion matrix of the predictions of clinical pregnancy using (a) conventional Gardner scoring assessment (control model), (b) blastocyst images (image‐only model; FiTTE), and (c) blastocyst images and clinical data (ensemble model). (d) Predictions of live births using blastocyst images. F1 score = 2/(recall^−1^+precision^−1^)

**FIGURE 4:**
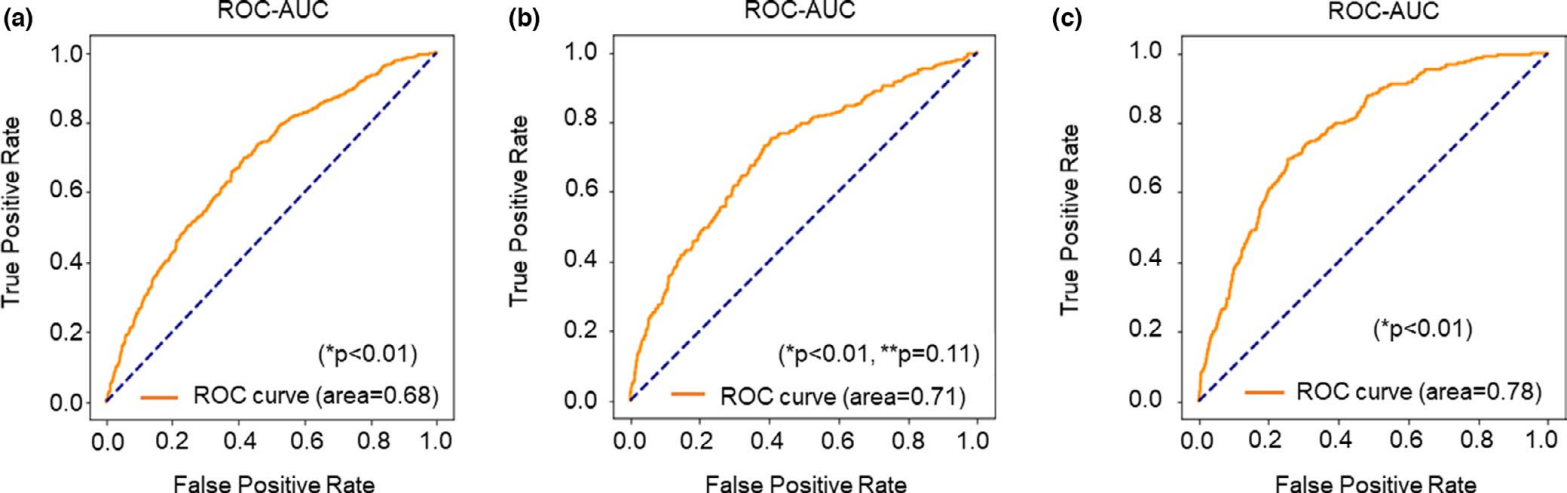
Receiver operator characteristic (ROC) curve constructed for predicting clinical pregnancy using (a) blastocyst images (image‐only model) and (b) blastocyst images plus clinical data (ensemble model). (c) ROC curve for predicting live birth from blastocyst images. *p value versus the control model (conventional Gardner scoring assessment). **p value, blastocyst images

Figure 3 reveals the confusion matrix of the prediction of clinical pregnancy. The prediction accuracy for positive pregnancy with fetal heartbeat using the conventional embryologist morphology assessment was calculated as 59.8% and was set as the control. The accuracy rate achieved by the image‐only AI model was improved at 62.7%. The accuracy rate achieved by the ensemble AI model was 65.2%, representing an improvement in prediction accuracy of 2.9% when evaluating against image‐only AI model, and 5.4% when evaluating against the visual inspection method performed by embryologists. Figure 4 shows the ROC analysis and represents the area under the curve (AUC) of the FiTTE to predict clinical pregnancy. AUC from the image‐only and ensemble models were 0.68 and 0.71, respectively (A, B), which are significantly better than those from the control model (*p* < 0.01). Similarly, AUC for predicting live birth using blastocyst images was 0.78 (C), which is also significantly better than that from the control model (*p* < 0.01). The difference between the AUC of image‐only (A) and ensemble models (B) is not statistically significant (*p* = 0.11).

**FIGURE 5:**
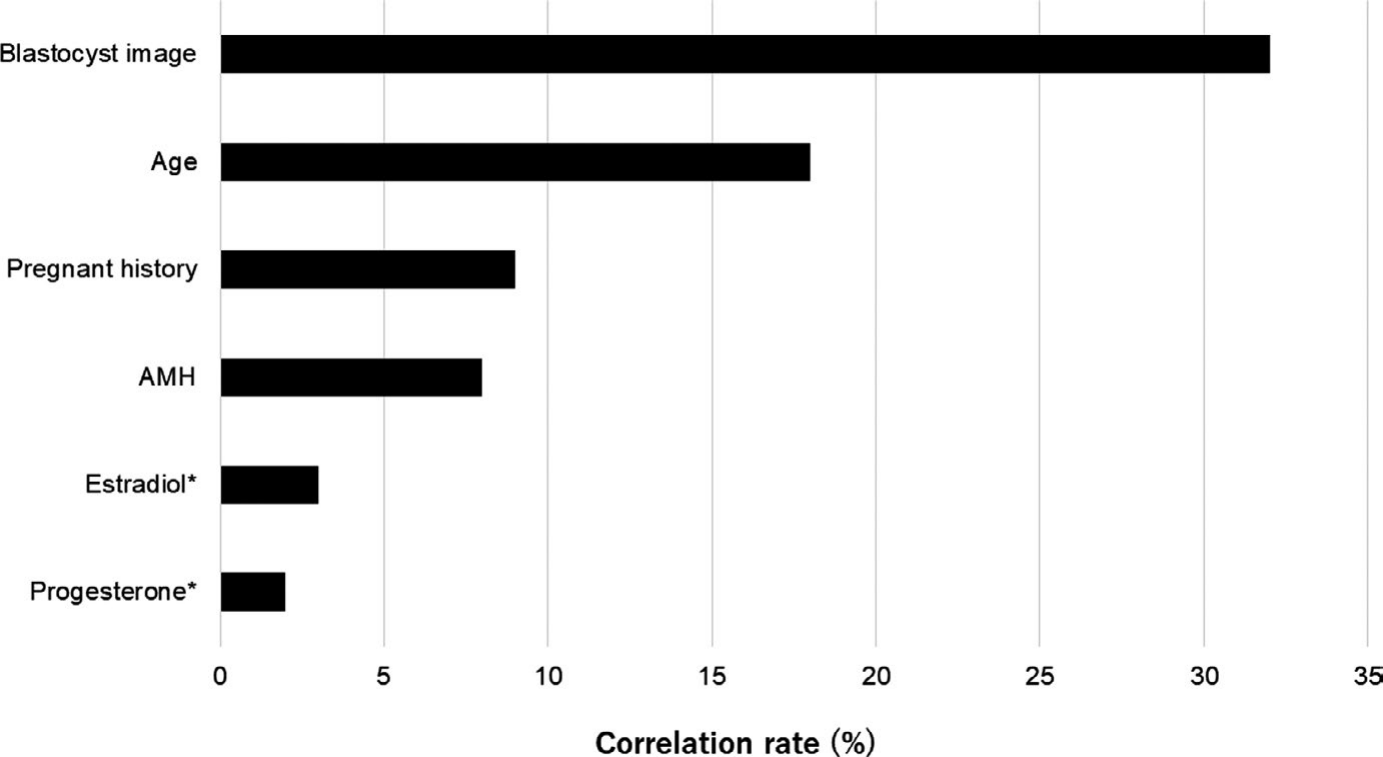
Variable importance analysis using the Shapley Additive Explanations (SHAP). The presented variables are the top six most important variables for predicting clinical pregnancy. *Serum estradiol and progesterone levels at the time of embryo transfer

Figure 5 represents the results of the variable importance analysis using the Shapley Additive Explanations (SHAP). It reveals that the ensemble model considered the blastocyst images as the most important factors for predicting clinical pregnancy, followed by age, pregnancy history, serum AMH, serum estradiol, and progesterone at the time of embryo transfer.

**FIGURE 6:**
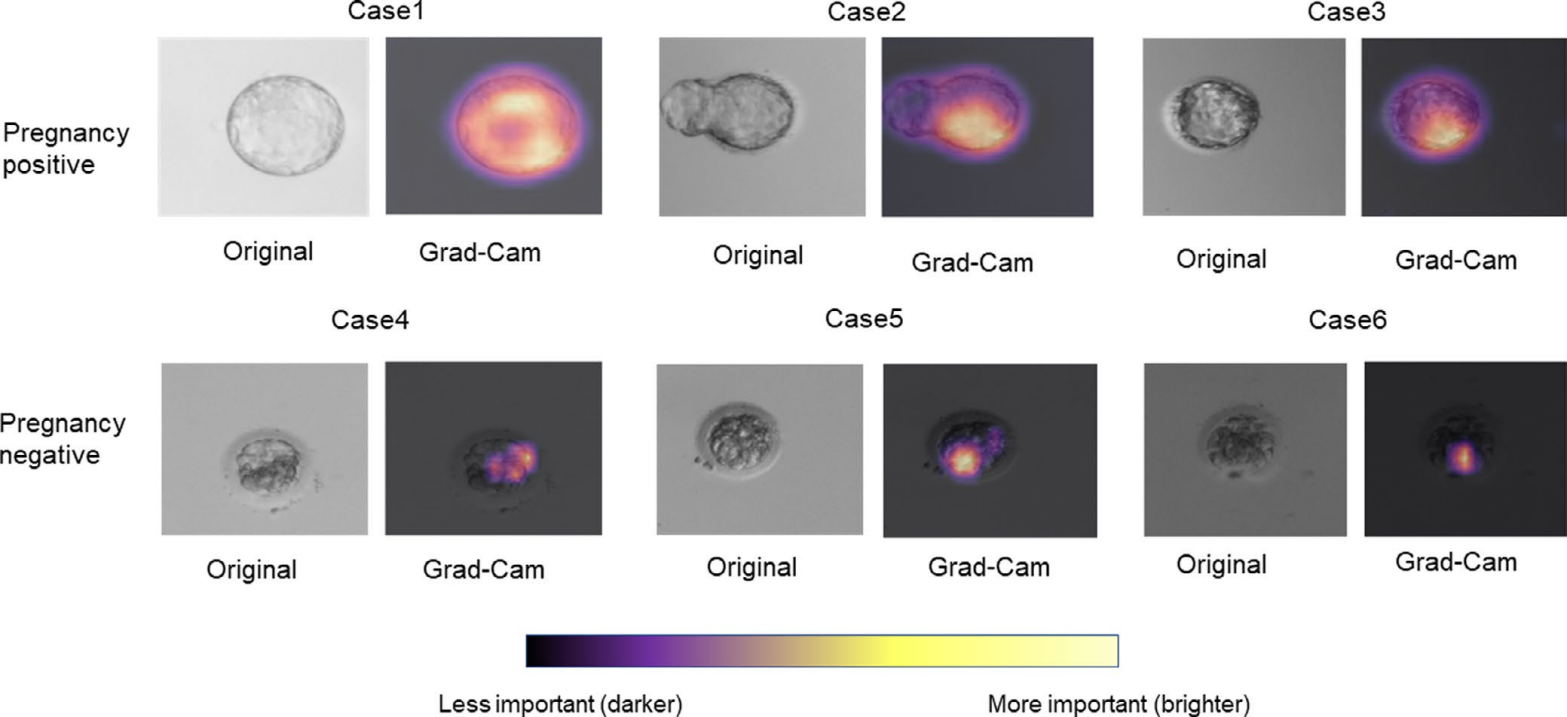
Gradient‐weighted class activation mapping (Grad‐CAM)‐assisted image identification for pregnancy prediction from blastocyst images. Cases 1–3 are blastocysts that resulted in positive pregnancies, and cases 4–6 are negative pregnancies. The images represent the original and those generated after applying the model with Grad‐CAM, which visualizes the class‐discriminative regions as the predictors of pregnancy

Figure 6 reveals the Grad‐CAM‐assisted image identification of pregnancy prediction from blastocyst images. Cases one to three involved blastocysts that resulted in positive pregnancies. Cases four to six resulted in negative pregnancies. When comparing positive pregnancy embryos with negative embryos, positive embryos showed overall bright colors by Grad‐CAM. In contrast, negative embryos showed small bright areas and large dark areas in the embryos. When positive pregnancy embryos were compared, case one revealed brighter colors than cases two and three. This shows that AI judged case one as having the highest pregnancy expectation of all the positive pregnancy embryos. Similarly, when the negative pregnancy embryos were compared, case six revealed the darkest color, meaning that AI judged case six as the least likely regarding pregnancy expectation.

## 4 DISCUSSION

This present study revealed that the AI system, FiTTE, can predict pregnancy expectation from blastocyst images more accurately than the conventional Gardner grading system. This can help physicians to select embryos for embryo transfer and also help embryologists to manually grade embryos under optical light microscope. Deep learning using AI has recently come into the spotlight for various medical imaging diagnosis applications such as detecting bone fracture, cancer, and diabetic retinopathy,^19^, ^20^, ^21^ with an accuracy of >90%. Unlike these studies, the difficult point of this field is that nobody can tell which embryo is viable or not. For example, when analyzing the bone fracture image, certain physicians can detect the bone fracture within the image, and the programmer can teach the AI the correct answer. Similarly, when detecting cancer or diabetic retinopathy, answers can be found in the images. However, in the field of predicting pregnancy, no definite answer can be obtained because viable embryos do not always result in pregnancy. This is because, besides embryos, various factors such as endometrial thickness, uterine myoma, endometritis, autoimmune system, and hormonal conditions can affect the outcome of clinical pregnancy. So far, several studies have developed AI models in the field of embryology. Filho et al. introduced a semi‐automatic grading system of human embryos, with accuracy rates ranging from 67%–92%.^22^ Similarly, Khosravi et al. developed an AI model that classifies blastocyst images using Gardner's classification.^23^ Although these reports achieved high accuracies, they set the end point as embryo classification and do not assess clinical pregnancy as an end point.

In the present study, FiTTE achieved an accuracy rate of 62.7% for clinical pregnancy from embryo images. We also confirmed that an ensemble model of blastocyst images and clinical data had a 2.5% improvement in accuracy for pregnancy prediction. The overall accuracy of 65.2% for prediction of clinical pregnancy was considered relatively high given that the theoretical maximum accuracy for prediction of pregnancy based on embryo evaluation is estimated to be <80%.^12^ Tran et al. reported that the deep learning model named IVY predicted the probability of fetal heartbeat from time‐lapse videos with an AUC of 0.93.^24^ Although this result revealed incredibly high reliability for predicting pregnancy, the datasets used for training and evaluation were only partly based on actual ground‐truth clinical pregnancy, indicating that a large proportion of predicted non‐viable embryos were not transferred. Therefore, the reported predictivity is not entirely relevant in the context of clinical application. To date, the Life Whisperer AI model reported by VerMilyea et al. is the biggest study that actually predicts clinical pregnancy,^9^ with a combined accuracy of 64.3%. This result is based on the same end point from our present study and revealed similar results as this present study. Considering the large number of datasets used in Life Whisperer and our present model, the maximum predictivity from this model will be between 60% and 70%.

To date, it is generally considered that the most accurate way to predict the viability of embryo is PGT. Indeed, the clinical outcomes of PGT are more accurate than those AI prediction models that the clinical pregnancy rate by euploid blastocyst is reported to be approximately 70%.^13^ However, PGT has several ethical problems related to its use such as mosaicism, embryo damage, and high cost. Therefore, a non‐invasive AI model that can predict clinical pregnancy with 65% accuracy is considered valuable.

This present study confirmed that of several variables, the blastocyst image was the best predictor of clinical pregnancy followed by age, pregnancy history, AMH, and estradiol and progesterone levels at the time of embryo transfer. This result seems reasonable since most clinicians consider embryo quality as the most important factor for pregnancy. In fact, prediction accuracy of FiTTE stratified in the age reveals that the accuracy was not different by age groups (Figures S1 and S2), indicating that the prediction of FiTTE can be used for patients with all reproductive ages. The present study shows a non‐significant improvement in prediction accuracy (2.5%) using an ensemble model as compared with an image‐only model. This result indicates that the present AI model does not fully assess other variables besides blastocyst images. This is presumably because gynecological images such as those obtained using uterine ultrasonography, hysteroscopy, hysterosalpingography, and magnetic resonance imaging were not included in the present study. In addition, the small sample size of ensemble model made it difficult to compare the result with image‐only model. Therefore, further studies regarding the use of additional data will be needed.

Another important aim of this study was the explanation of AI prediction. Physicians and embryologists are obliged to explain the results about embryos to patients before transfer, indicating that physicians and embryologists must understand why the AI model derived the results. To date, several studies have already presented great opportunities for applying deep learning in the medical field. Cheng et al. showed the visualization of hip fractures on plain pelvic radiographs.^20^ Similarly, Burduja et al. presented the intracranial hemorrhage detection system with Grad‐CAM visualization.^25^ These studies revealed the usefulness of Grad‐CAM by providing useful explanations for its predictions. In this present study, we revealed that our AI system considered the well‐cleaved area in the blastocyst as characteristic of high pregnancy expectation. In contrast, areas with unequal cleavage and/or with fragmentation were considered characteristic of low pregnancy expectation. Roughly, these characteristics were similar to those of conventional Gardner's classification. Therefore, it is comprehensible why AI concluded pregnancy expectation in individual embryos. This will help physicians to explain the result of FiTTE to patients. In some cases, we found that the visual characteristic patterns by FiTTE were different from those of conventional Gardner's classification. Although such cases may confuse clinicians regarding interpreting the result, these differences may provide new insight into the developmental pattern of embryos in the future.

This study is not without limitations. First, this study was based on the analysis of static blastocyst images. Although the use of still images in an AI system in the IVF laboratory is of importance since not all laboratories are equipped with time‐lapse, dynamic analysis of embryos by analyzing time‐lapse images is needed for a more accurate analysis. Second, this study only included Asian populations, mostly Japanese, who have different characteristics from other races; therefore, it may be difficult to apply the present FiTTE system to the entire cohort of patients receiving ART. Finally, this study was a non‐randomized, retrospective study, with a limited sample size. Moreover, since the embryos were selected for embryo transfer by humans, the false‐positive rate of the AI model might have been underrepresented. Therefore, a larger prospective study is necessary to establish the true benefit of FiTTE in the field of ART.

In conclusion, we invented the novel AI system named FiTTE, which could predict the probability of clinical pregnancy using blastocyst images secondary to single embryo transfer more precisely than the conventional Gardner scoring assessment. Although the prediction accuracy was slightly improved in the model of images plus clinical data, blastocyst images had the greatest impact on the prediction. FiTTE could also provide visual explanation of the AI prediction using colored blastocyst images.

## CONFLICT OF INTEREST

This study was funded in part by the Kobe Medical Industry Development Project, which is a public grant providing support in the form of research materials. The funders had no role in the study design, data collection and analysis, decision to publish, or preparation of the manuscript. Isao Miyatsuka and Le My An are employees of NextGem Inc. The equipment and programs have been jointly provided by NextGem Inc.

## ETHICAL APPROVAL

The statement of approval from the Institutional Review Board: This study was approved by the Ethical Committee of Hanabusa Women's Clinic consists of members chosen by our institute and third party medical institute (approval number; 2019–06).

## HUMAN RIGHTS STATEMENT AND INFORMED CONSENT

All patients were well‐informed, and written informed consent was obtained prior to the treatment period.

## ANIMAL RIGHTS

This article does not contain any studies with animal subjects performed by the any of the authors.

## Supporting information

Fig S1

Fig S2

Supplementary Material
