# Supplementary figures and images for: A novel system based on artificial intelligence for predicting blastocyst viability and visualizing the explanation
Source: Reprod Med Biol. 2022 Feb 7;21(1):e12443. doi: 10.1002/rmb2.12443 (PMC8967284; doi:10.1002/rmb2.12443)

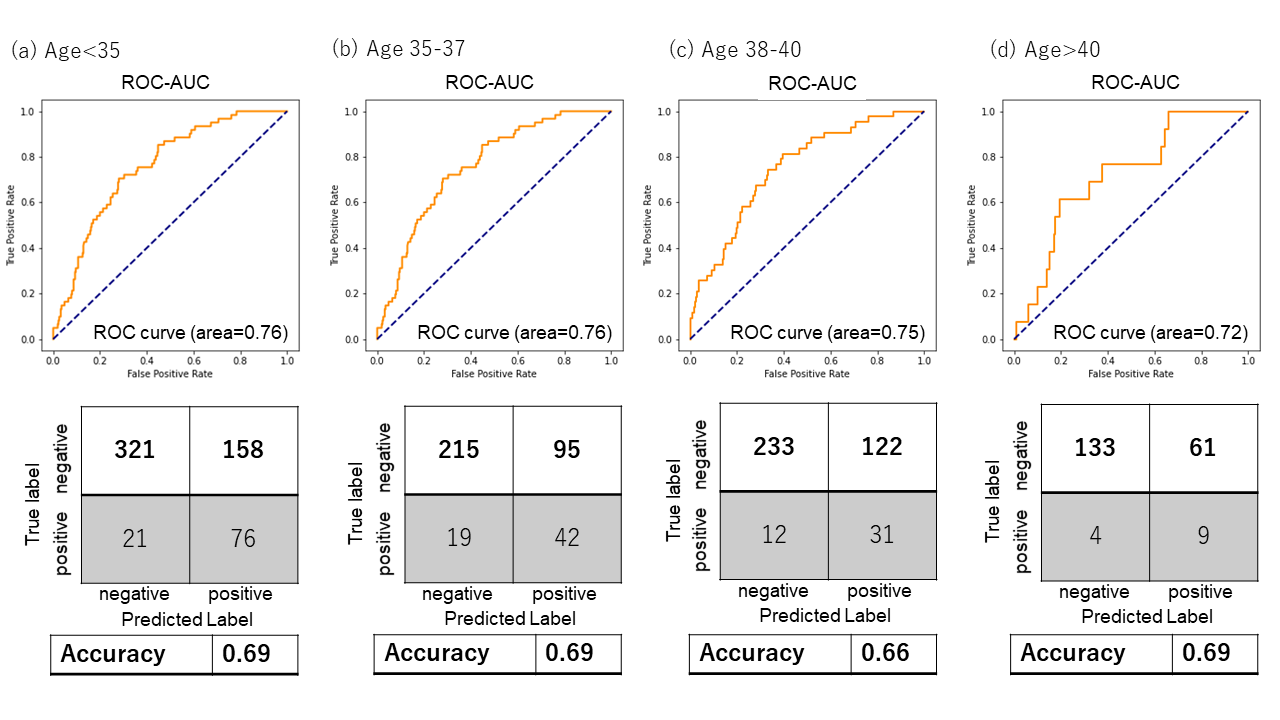

Supplement: Supplementary file 1 — Fig S1 [file RMB2-21-e12443-s003.tif]

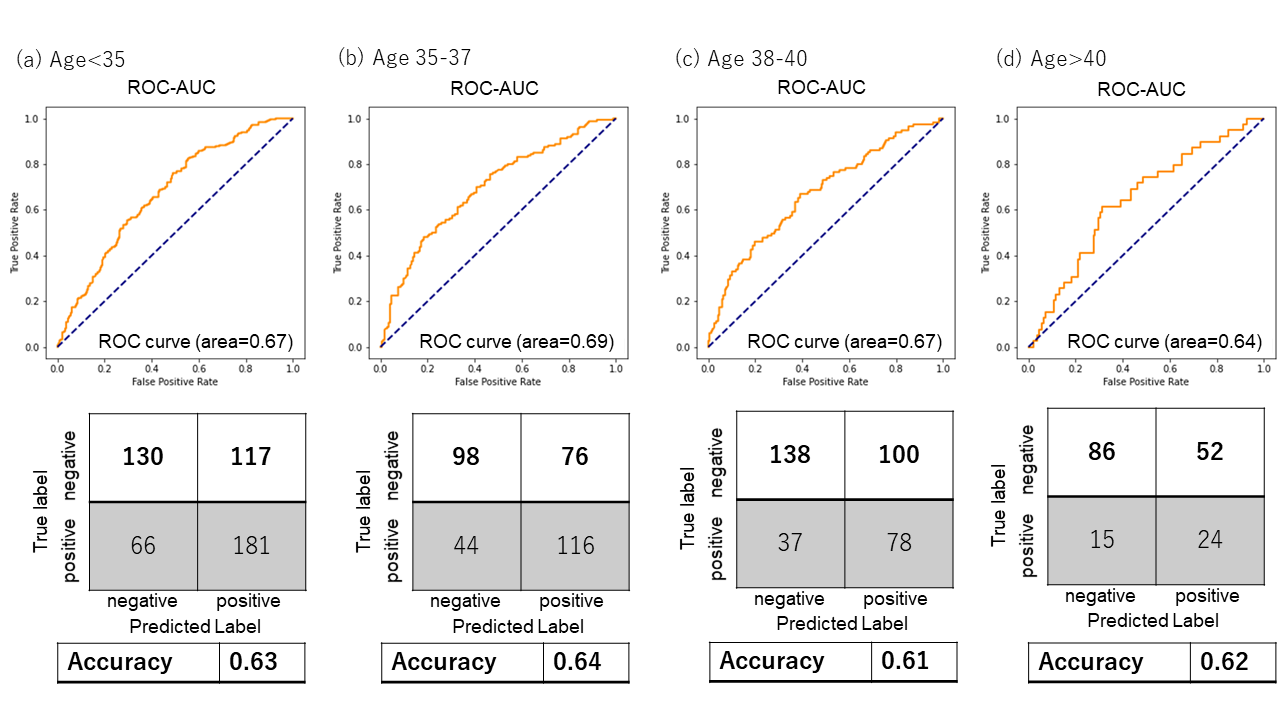

Supplement: Supplementary file 2 — Fig S2 [file RMB2-21-e12443-s002.tif]
