# Supplementary material for: A novel system based on artificial intelligence for predicting blastocyst viability and visualizing the explanation
Source: Reprod Med Biol. 2022 Feb 7;21(1):e12443. doi: 10.1002/rmb2.12443 (PMC8967284; doi:10.1002/rmb2.12443)
Supplement: Supplementary file 3 — Supplementary Material [file RMB2-21-e12443-s001.docx]

Supplemental Figure 1. Clinical pregnancy prediction outcomes stratified in the age using FiTTE (image-only model). (a) Age < 35; 494 cases, accuracy 0.63. (b) Age 35 – 37; 334 cases, accuracy 0.64. (c) Age 38 – 40; 353 cases, accuracy 0.61. (d) Age > 40; 177 cases, accuracy 0.62.

Supplemental Figure 2. Live birth prediction outcomes stratified in the age using FiTTE (image-only model). (a) Age < 35; 576 cases, accuracy 0.69. (b) Age 35 – 37; 371 cases, accuracy 0.69. (c) Age 38 – 40; 398 cases, accuracy 0.66. (d) Age > 40; 207 cases, accuracy 0.69.
